# Efficient Synthesis of Purine Nucleoside Analogs by a New Trimeric Purine Nucleoside Phosphorylase from *Aneurinibacillus migulanus* AM007

**DOI:** 10.3390/molecules25010100

**Published:** 2019-12-26

**Authors:** Gaofei Liu, Tiantong Cheng, Jianlin Chu, Sui Li, Bingfang He

**Affiliations:** 1 College of Biotechnology and Pharmaceutical Engineering, Nanjing Tech University, Nanjing 211800, China; 201762100110@njtech.edu.cn (G.L.); Cttdk123@163.com (T.C.); 2 School of Pharmaceutical Sciences, Nanjing Tech University, Nanjing 211800, China; 1303160204@njtech.edu.cn

**Keywords:** trimeric nucleoside phosphorylase, thermostability, purine nucleoside analogs, enzyme ratio

## Abstract

Purine nucleoside phosphorylases (PNPs) are promising biocatalysts for the synthesis of purine nucleoside analogs. Although a number of PNPs have been reported, the development of highly efficient enzymes for industrial applications is still in high demand. Herein, a new trimeric purine nucleoside phosphorylase (*Am*PNP) from *Aneurinibacillus migulanus* AM007 was cloned and heterologously expressed in *Escherichia coli* BL21(DE3). The *Am*PNP showed good thermostability and a broad range of pH stability. The enzyme was thermostable below 55 °C for 12 h (retaining nearly 100% of its initial activity), and retained nearly 100% of the initial activity in alkaline buffer systems (pH 7.0–9.0) at 60 °C for 2 h. Then, a one-pot, two-enzyme mode of transglycosylation reaction was successfully constructed by combining pyrimidine nucleoside phosphorylase (*Bb*PyNP) derived from *Brevibacillus borstelensis* LK01 and *Am*PNP for the production of purine nucleoside analogs. Conversions of 2,6-diaminopurine ribonucleoside (**1**), 2-amino-6-chloropurine ribonucleoside (**2**), and 6-thioguanine ribonucleoside (**3**) synthesized still reached >90% on the higher concentrations of substrates (pentofuranosyl donor: purine base; 20:10 mM) with a low enzyme ratio of *Bb*PyNP: *Am*PNP (2:20 μg/mL). Thus, the new trimeric *Am*PNP is a promising biocatalyst for industrial production of purine nucleoside analogs.

## 1. Introduction

Up to now, nucleoside analogs have been significant sources of antiviral and anticancer drugs [1,2,3], among which purine nucleoside analogs are widely used for the treatment of leukemia [4,5] and are also important precursors of several drugs [6,7]. To date, most purine nucleoside analogs are mainly synthesized by chemical methods [8,9,10]. However, biotechnological or enzymatic synthesis of purine nucleosides has become an attractive alternate to chemical synthesis, due to its higher chemo-, regio-, and stereoselectivity and greener reaction conditions [11]. Moreover, enzymatic synthesis of nucleosides circumvented multiple steps of activation, protection, and deprotection of functional groups [12,13], demonstrating great potential for the industrial production.

Nucleoside phosphorylases (NPs) catalyze the reversible cleavage of the glycosidic bond of (deoxy)ribonucleosides in the presence of inorganic orthophosphate (Pi) to generate a nucleobase and α-d-(deoxy) ribose-1-phosphate [14]. NPs include pyrimidine NPs (PyNPs, EC 2.4.2.2) and purine NPs (PNPs, EC 2.4.2.1). PyNPs show substrate specificity for the phosphorolysis of pyrimidine nucleosides, while PNPs are specific for purine nucleoside substrates [15]. For the effective formation of purine nucleosides, PyNPs and PNPs are usually combined in a one-pot, two-enzyme reaction system. For example, PNP from *T. thermophilus* and PyNP from *Geobacillus thermoglucosidasius* were used as biocatalysts to synthesize 2,6-diaminopurine ribonucleoside, which obtained a yield of 95.3% using uridine and 2,6-diaminopurine (2:1 mM) as substrates [16]. 

PNPs play a more important role in this system, which determines the yield of purine nucleosides. Currently, most PNPs are divided into trimeric PNPs and hexametric PNPs based on molecular mass, protein structure, or substrate specificity. Trimeric PNPs are isolated mainly from mammalian cells, including human erythrocytes [17] and bovine brain cells [18], which have a subunit molecular mass of approximately 31 kDa and display specific activity toward guanosine and inosine. The hexametric PNPs are mostly isolated from bacterial species like *Salmonella typhimurium* [19] and *Aeromonas hydrophila* [20], which exhibit a phosphorolysis activity against adenosine and some derivatives, and have a subunit molecular mass of approximately 26 kDa. Although trimeric PNPs have good thermal stability, they have narrower substrate specificity. Therefore, only a few trimeric PNPs have been reported for purine nucleoside synthesis, which limits its application in industrial production. Therefore, it is still very urgent to screen and discover new trimeric PNPs.

The high price of enzymes increases the production cost in the enzyme-catalyzed synthesis industry. An appropriate enzyme ratio can not only increase the yield of the product, but also reduce the use of one enzyme, thus reducing the production cost. Zhou et al. used *Gt*PNP and *Tt*PyNP (enzyme ratio; 2:1) to synthesize purine nucleoside analogs with high yield [16]. Subsequently, their lab also reported that purine nucleosides with a higher yield were also synthesized by the combination of *Tt*PyNP and *Gt*PNP (enzyme ratio; 1:1) [21]. Therefore, in order to reduce the production cost, the optimization of the enzyme ratio is also necessary.

In this study, a trimeric PNP was cloned from *Aneurinibacillus migulanus* AM007 (CCTCC NO. M2016404) and heterologously expressed in *Escherichia coli* BL21(DE3). This enzyme named *Am*PNP was characterized. Interestingly, it is different from trimeric PNPs in the phosphorolysis activity of adenosine. Highly efficient synthesis of purine nucleosides analogs was successfully achieved in the one-pot, two-enzyme transglycosylation reaction catalyzed by *Am*PNP and PyNP derived from *Brevibacillus borstelensis* LK01 (*Bb*PyNP) [22]. In particular, the ratio of *Bb*PyNP and *Am*PNP was optimized for the one-pot, two-enzyme reaction, greatly reducing the production cost.

## 2. Results and Discussion

### 2.1. Sequence Analysis, Cloning, Expression, and Purification of AmPNP

In this study, in order to screen the trimeric PNP with thermostability, *Bs*PNP (GenBank: WP_015714207) from *B. subtilis* was selected as a template to mine thermostable PNPs among the genomes of 24 thermostable strains in our lab [23]. After preliminary experiments, the PNP from *A. migulanus* AM007, with higher enzyme activity toward purine nucleosides, was selected as a potential biocatalyst for the synthesis of purine nucleoside analogs. The strain *A. migulanus* AM007 was deposited at the CCTCC (accession number of CCTCC NO. M2016404).

The open-reading frame (ORF) of the *Am*PNP gene (813 bp) corresponded to 270 amino acid residues. The amino acid sequence of *Am*PNP from *A. migulanus* AM007 was analyzed with sequence alignment (Supplementary Material, Table S1), which had a high identity of 66.1% with trimeric *Bs*PNP from *B. subtilis*. The amino acid sequence of *Am*PNP showed 46.4%, 45.3%, 14.7%, and 13.1% identity with the sequences of PNPs from humans, bovine, *E. coli*, and *Aeromonas hydrophila*, respectively, showing that it is more similar to trimeric PNPs.

**Figure 1:**
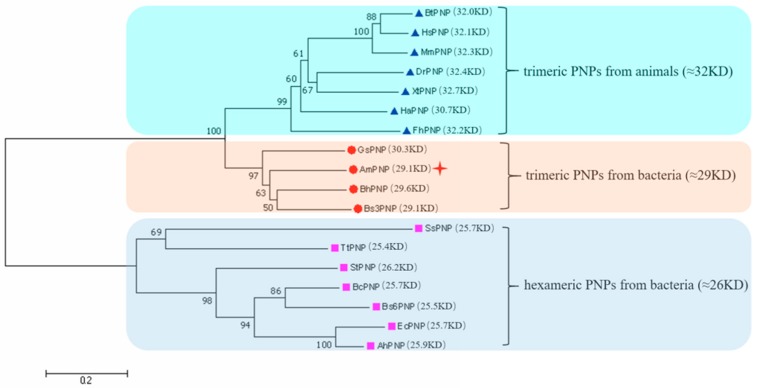
Phylogenetic analysis of purine nucleoside phosphorylases (PNPs). *Ah*PNP (GenBank:WP_049045821) from *Aeromonas hydrophila*; *Bc*PNP (GenBank:WP_098273438) from *Bacillus cereus*; *Bh*PNP (GenBank: WP_010897696) from *Bacillus halodurans*; BtPNP (GenBank: AAX46392) from *Bos taurus*; *Bs*3PNP (GenBank: WP_015714207) from *Bacillus subtilis*; *Bs*6PNP (GenBank: WP_044428467) from *Bacillus subtilis*; DrPNP (GenBank: NP_998476) from *Danio rerio*; *Ec*PNP (GenBank: AAN83888) from *Escherichia coli*; FhPNP (GenBank: XP_012736771) from *Fundulus heteroclitus*; *Gs*PNP (GenBank: WP_053414649) from *Geobacillus stearothermophilus*; HaPNP (GenBank:KFP95377) from *Haliaeetus albicilla*; HsPNP (GenBank: NP_000261) from *Homo sapiens*; MmPNP (GenBank: NP_038660) from *Mus musculus*; *Ss*PNP (GenBank: WP_009988635) from *Sulfolobus solfataricus*; *St*PNP (GenBank: WP_011681178) from *Streptococcus thermophilus*; *Tt*PNP (GenBank: WP_096412123) from *Thermus thermophilus*; and XtPNP (GenBank: NP_001006720) from Xenopus tropicalis.

A phylogenetic tree was created based on the sequences of *Am*PNP and other reported trimeric and hexametric PNPs (Figure 1). The phylogenetic tree indicates that the trimeric PNPs from the bacterial species were a special subfamily of PNPs that displayed a different branch from the trimeric PNPs derived from animals. This implies that trimeric PNPs from bacterial species are different from traditional trimeric PNPs from animals in structure and function. 

**Figure 2:**
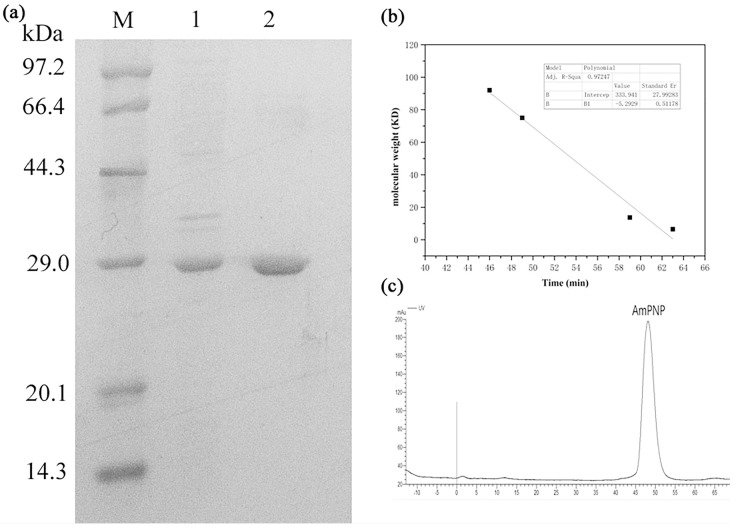
Molecular weight analysis of *Am*PNP. (**a**) Dodecyl sulfate-polyacrylamide gel electrophoresis (SDS-PAGE) analysis of purified *Am*PNP. Lane M: marker; Lane 1: crude enzymes; Lane 2: enzyme of *Am*PNP purified by Ni^2+^ column. (**b**) Analysis of *Am*PNP by gel filtration chromatography. The condition was conducted as following: an ÄKTA pure system and a column Superdex 200 Increase 10/300 GL were used. The standard proteins and the purified *Am*PNP sample were dissolved in the buffer (0.01 M phosphate buffer, 0.14 M NaCl, pH 8.0) with the final concentration of 2 mg/mL, respectively. For analysis, the flow rate was set at 0.3 mL/min and the inject volume was 500 μL.

The gene encoding *Am*PNP (GenBank accession number: MH992394) from *A. migulanus* AM007 was successfully expressed in *E. coli* BL21(DE3). SDS-PAGE analysis of the recombined *Am*PNP revealed a thick band of approximately 29 kDa corresponding to the possible subunit molecular mass of the enzyme. The 6*His-tagged (N-terminal) recombinant *Am*PNP was purified with Ni^2+^-affinity chromatography from the cell extract, and purified *Am*PNP was obtained with high purity (Figure 2a). The molecular weight of the purified *Am*PNP was about 87 kDa, as analyzed with size-exclusion chromatography (Figure 2b,c). According to the SDS-PAGE analysis and gel-filtration chromatography results, the *Am*PNP obtained from *A. migulanus* AM007 was a trimeric protein complex, which was consistent with the observation reported in the phylogenetic tree. 

### 2.2. Specific Activities of AmPNP Toward Various Purine Nucleosides

A variety of purine nucleosides were used for the exploration of the substrate spectrum of *Am*PNP (Table 1). The highest activity was obtained for inosine among purine nucleosides. According to previous literature [14], trimeric PNPs did not display specific activity toward adenosine. Interestingly, the trimeric *Am*PNP could cleave adenosine by phosphorolysis. This also suggested that the substrate specificity of the trimeric PNPs from bacterial species were different from trimeric PNPs from animals. In addition, the K_cat_/K_m_ values (Table 2) of *Am*PNP for inosine and guanosine were considerably higher than other PNPs, such as the PNP from *Aeropyrum pernix* K1 [24], indicating that it could serve as a useful biocatalyst in biochemistry and industrial biotechnology.

### 2.3. Characterization of AmPNP

**Scheme 1:**
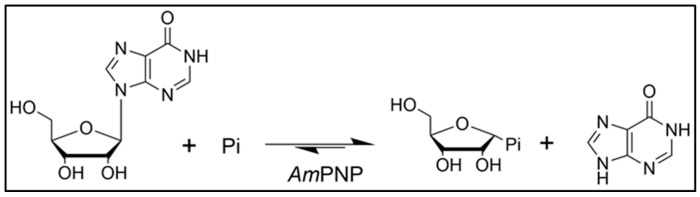
Phosphorylation of inosine with *Am*PNP.

**Figure 3:**
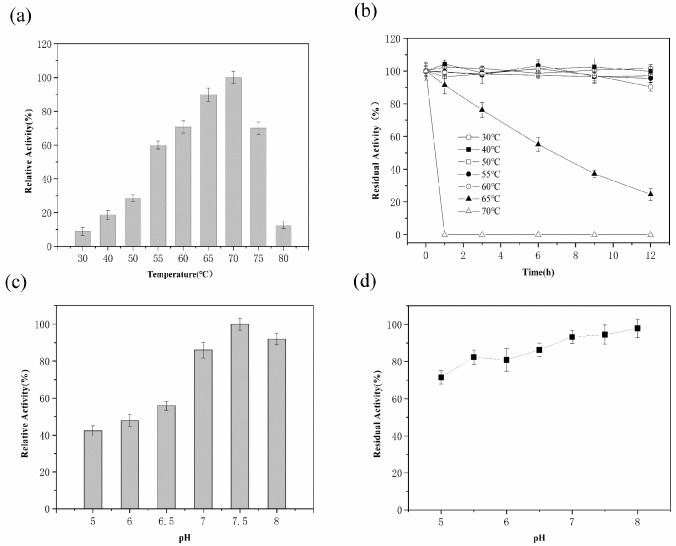
Effects of temperature and pH on the activity and stability of *Am*PNP. (**a**) Effect of temperature on the activity of *Am*PNP. The highest activity was set to 100%. (**b**) Effects of temperature on stability of *Am*PNP. The initial activity was set to 100%. (**c**) Effect of pH on the activity determined by assaying the activity at 60 °C in 10 mM Na_2_HPO_4_/KH_2_PO_4_ buffer. The highest activity was set to 100%. (**d**) Effect of pH on stability determined by assaying the residual activity of *Am*PNP after 2 h at 60 °C and different pH values in 10 mM Na_2_HPO_4_/KH_2_PO_4_ buffer. The highest activity was set to 100%. The activity of *Am*PNP was determined in 10 mM Na_2_HPO_4_/KH_2_PO_4_ buffer (pH 8.0) with 10 mM inosine.

The purified *Am*PNP was used to determine its biochemical properties. The *Am*PNP exhibited maximum phosphorolysis activity (Scheme 1) at a temperature of 70 °C (Figure 3a). The enzyme retained nearly 100% of its activity below 55 °C for 12 h (Figure 3b), and the half-life period of *Am*PNP was 78.6 h, 37.3 h, and 6.9 h at temperatures of 55 °C, 60 °C, and 65 °C, respectively (Supplementary Material, Table S2). Overall, the *Am*PNP showed good thermostability, which was useful for industrial purine nucleosides synthesis done at a relatively high temperature to increase the solubility of substrate. The highest phosphorolysis activity of *Am*PNP was observed at a pH of 7.5 (Figure 3c). The enzyme retained nearly 100% of the initial activity in the alkaline buffer systems (pH 7.0–8.0) at 60 °C for 2 h (Figure 3d). The broad range of pH stability by *Am*PNP is facilitated to adjust the reaction pH with two-enzyme reaction. Therefore, the application of *Am*PNP in industrial production has become more promising. 

### 2.4. Effect of Temperature and pH on Two-Enzyme Biosynthesis of 2-Amino-6-chloropurine Ribonucleoside *(**2**)*

**Scheme 2:**
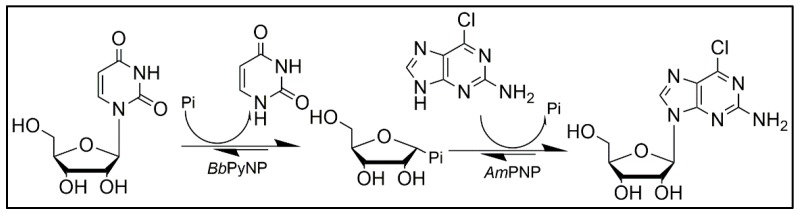
Enzymatic synthesis of 2-amino-6-chloropurine ribonucleoside.

In order to efficiently synthesize halogenated purine nucleoside, it was necessary to conduct the transglycosylation reaction of *Am*PNP as the reported two-enzyme system [16]. We previously reported that *Bb*PyNP from *Brevibacillus borstelensis* LK01 catalyzed the phosphorolysis of thymidine, 2′-deoxyuridine, uridine, and 5-methyuridine [22]. What is more, the pH and thermostability of *Bb*PyNP were nearly similar to those of *Am*PNP from *A. migulanus* AM007. Therefore, it is more favorable for synthesis of purine nucleoside by combining *Bb*PyNP and *Am*PNP (Scheme 2). 

**Figure 4:**
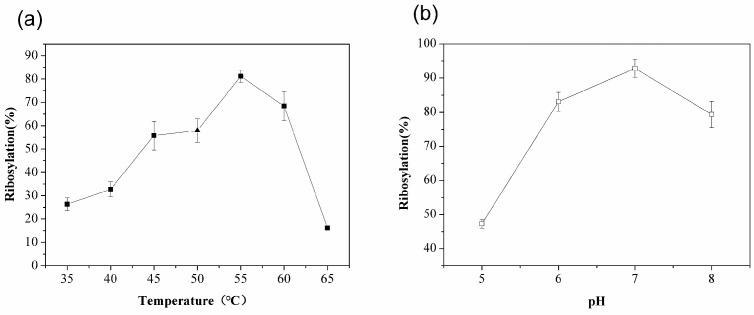
Effect of temperature and pH on biosynthesis of 2-amino-6-chloropurine ribonucleoside *Bb*PyNP: *Am*PNP in a ratio of 2:20 μg/mL. The reaction mixture (1.0 mL) contained 20 mM uridine and 10 mM 2-amino-6-chloropurine. (**a**) The reaction was conducted in a thermostatic oscillator at a pH of 8.0 (20 mM Na_2_HPO_4_/KH_2_PO_4_) with temperature ranging from 30 °C to 65 °C for 36 h. (**b**) The reaction was carried out at a temperature of 55 °C for 60 h in 20 mM Na_2_HPO_4_/KH_2_PO_4_ buffer systems with pH ranging from 5.0 to 8.0.

Using pyrimidine nucleosides as donors, several purine nucleosides, including 2-amino-6-chloropurine ribonucleoside (**2**), were synthesized with *Bb*PyNP and *Am*PNP (ratio of 0.1:1) and the conditions of the one-pot, two-enzyme reaction were studied (Figure 4). The optimum temperature for the *Am*PNP and *Bb*PyNP enzymes was approximately 55 °C, and the yield of the 2-amino-6-chloropurine ribonucleoside (**2**) product reached approximately 81.2% (Figure 3a). As shown in Figure 3b, a conversion >70% was achieved from pH 6.0 to 8.0 in the one-pot, two-enzyme reaction. The maximum 2-amino-6-chloropurine ribonucleoside (**2**) product formation reached approximately 92.8% at pH 7.0 and 55 °C under relatively high substrate concentrations (ratio of uridine to purine of 20:10 mM) (Supplementary Material, Figure S1).

### 2.5. Effect of BbPyNP and AmPNP Ratio on the Biosynthesis of 2-Amino-6-chloropurine Ribonucleoside *(**2**)*

**Figure 5:**
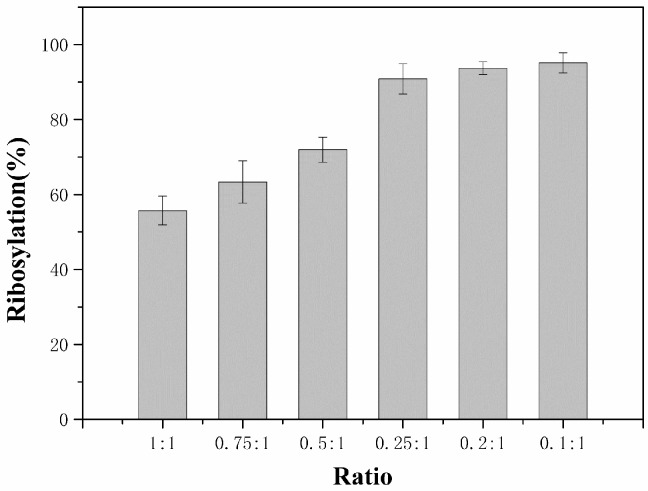
Effect of the ratio of *Bb*PyNP: *Am*PNP on biosynthesis of 2-amino-6-chloropurine ribonucleoside. Process for synthesis of 2-amino-6-chloropurine ribonucleoside was shown in Scheme 2. Using 20 mM uridine and 10mM 2-amino-6-chloropurine, the reaction mixture (1.0 mL) contained purified *Bb*PyNP (85 IU/mg for uridine) and purified *Am*PNP (83 IU/mg) at different ratios (the concentration of *Am*PNP was set at 20 μg/mL). The reaction was carried out at pH 7.0 (20 mM Na_2_HPO_4_/KH_2_PO_4_) and a temperature of 55 °C for 36 h, followed by analysis.

In the previous literature, Zhou et al. used *Gt*PNP (0.2 mg/mL) and *Tt*PyNP (0.1 mg/mL) to synthesize purine nucleoside analogs with a higher yield [16]. Subsequently, their lab also reported that purine nucleosides with higher yields were also synthesized by the combination of *Tt*PyNP (0.05 mg/mL) and *Gt*PNP (0.05 mg/mL) [21]. Purine nucleosides were both synthesized with a high yield under different enzyme ratios. In this context, the optimization of the enzymes ratio is also necessary to reduce the cost of production in the industry. It was found that a low ratio of *Bb*PyNP: *Am*PNP (2:20 μg/mL) also resulted in the higher yield (95.1%) of the 2-amino-6-chloropurine ribonucleoside (**2**) product (Figure 5). The phosphorolysis of uridine as a ribose donor produces the α-d-ribose-1-phosphate and free uracil in a phosphate buffer with *Bb*PyNP. α-d-ribose-1-phosphate is an unstable compound [25], and the excessive compound will decompose. In the higher ratio of *Bb*PyNP for *Am*PNP, transglycosylation cannot catch up with decomposition of ribose-1 alpha-phosphate. A low ratio of *Bb*PyNP: *Am*PNP (2:20 μg/mL) would reduce the usage of the PyNP by 25 times than previous reported in [21], which could give a reduction in cost and show the important value of producing it in the industry. 

### 2.6. Enzymatic Synthesis of Various Purine Nucleoside Analogs 

Various purine nucleoside analogs could be biosynthesized by coupling *Am*PNP and *Bb*PyNP under optimum conditions for the one-pot, two-enzyme cascade reaction at yields ranging from 3.4% to 98.3% (Table 2). 2-amino-6-chloropurine nucleoside was selected for confirmation and identified by the NMR spectrum (Supplementary Material Figures S21 and S22). NMR spectroscopic characterization of the product 2-amino-6-chloropurine ribonucleoside (**2**) was identical to the corresponding product when compared with the report [26]. 

In the previous literature, various purine nucleoside analogs were also synthesized by NPs (Table 3). For 2,6-diaminopurine ribonucleoside (**1**), the PNP from *A. pernix* K1 was reported to synthesize the product (**1**) at an approximate yield of 60% [27]. The PNP from *T. thermophilus* (*Tt*PNP) and the PyNP from the *G. thermoglucosidasius* (*Gt*PyNP) [16] synthesized product (**1**) with the highest yield of 95.3% at a lower substrate concentration of 2 mM uridine and 1 mM 2,6-diaminopurine. For 2-amino-6-chloropurine ribonucleoside (**2**), using PNP from *A. hydrophila*, product (**2**) was synthesized at a yield of 93% in the presence of 0.42 mM 7-methylguanosine iodide and 0.14 mM 2-amino-6-chloropurine [26]. In comparison, using uridine as donor, the conversion of product (**1**) synthesized by the combination of *Am*PNP and *Bb*PyNP reached higher than 98.3% (9.8 mM) at higher concentrations of 20 mM uridine and 10 mM 2,6-diaminopurine. Other products of ribo-purine nucleoside analogs were obtained at a yield of 95.1% for product (**2**) and 92.3% for product (**3**), corresponding to a nucleoside production level of 9.5 mM and 9.2 mM, respectively. Although the concentrations of substrates were higher than the reports, the conversions of both product (**1**) and product (**2**) synthesized by the combination of *Am*PNP and *Bb*PyNP still reached more than 95%. Also, some halogenated purine 2′-deoxyribonucleosides were synthesized by the combination of *Am*PNP and *Bb*PyNP. Specifically, 2-Chloro-2′-deoxyadenosine (Cladribine) was formed at a yield of 60.4%, and an antiviral drug ribavirin was also formed with a yield of 73.7% with the transglycosylation reaction. These results indicate that *Am*PNP is an excellent biocatalyst for the industrial production of purine nucleoside analogs.

## 3. Materials and Methods

### 3.1. Materials

Nucleosides and purine bases were purchased from Aladdin (Shanghai, China). Other chemicals obtained were of analytical grade. Solvents used for high-performance liquid chromatography (HPLC) were of HPLC grade. The One Step Cloning Kit was purchased from Novoprotein (Shanghai, China). The vector pET-28a (+) and strain *E. coli* BL21(DE3) were obtained from Novagen (New haven, CT, USA) for plasmid amplification or protein expression. The restriction enzymes and T4 DNA ligase were purchased from Takara (Kyoto, Japan).

### 3.2. Cloning, Expression, and Purification of AmPNP from A. migulanus AM007

The mesophilic strain *A. migulanus* AM007 was previously isolated and deposited at the China Center for Type Culture Collection (CCTCC, Wuhan, China) with the accession number CCTCC NO. M2016404. The gene encoding PNP from A. *migulanus* AM007 was amplified, and then cloned into pET-28a (+) using the *Nhe* I and *BamH* I restriction enzymes. The primers used were as following: forward, 5′-CGCGGCAGCCATATGGCTAGCATTGCAGCGCATATTTCTG -3′ with the *Nhe I* restriction site (underlined), and reverse, 5′-ACGGAGCTCGAATTCGGATCCTTATAATTTTTGAATAACAGCTTTT-3′ with the *BamH I* restriction site (underlined). The recombinant plasmids were constructed using the ClonExpress II One Step Cloning Kit, and then they were transformed into *E. coli* BL21(DE3) (Novagen). Cell growth was carried out at 37 °C. When the optical density at 600 nm (OD600) of the cell suspension reached 0.6, and after which the temperature was lowered to 20 °C, isopropyl β-d-1-thiogalactopyranoside (IPTG) was added with a final concentration of 1 mM for the enzyme expression. The cells were harvested by centrifugation (12,000 rpm, 4 °C, 10 min) after induction for 8 h. The cell pellets were resuspended in phosphate-buffered saline (PBS; Na_2_HPO_4_/KH_2_PO_4_, 50 mM, pH 8.0) and subjected to ultrasonic fragmentation on ice using an GA92-IID ultrasonicator (Wuxi Shangjia Biotechnology Co., Ltd., Jiangsu, China). The solution of the recombinant protein with an N-terminal 6*His-tag was obtained by centrifugation with the same conditions above, and then was purified with Ni^2+^-affinity chromatography. *Am*PNP was analyzed by sodium dodecyl sulfate polyacrylamide gel electrophoresis (SDS-PAGE) and its molecular mass was determined using gel-filtration chromatography on a Superdex 200 Increase 10/300 GL column (GE Healthcare, Buckinghamshire, UK). Protein concentrations were determined with bicinchoninic acid (BCA) Protein Assay Kit (Zhongke rRuitai, Biological Technology Co., Ltd., Beijing, China). 

### 3.3. Analysis of Enzyme Activity and Substrate Specificity

One international unit of enzyme activity (IU) was defined as the amount of enzyme required to convert 1.0 μmol of inosine per minute at 50 °C and a pH of 8.0 in 10 mM Na_2_HPO_4_/KH_2_PO_4_ with 10 mM inosine as substrate. The reaction was performed by adding 50 μL of enzyme (15 μg/mL) to 1.0 mL of the reaction mixture. Regular samples (every 5 min) were taken, and 50 μL of the reaction mixture was immediately added to 950 μL methanol to stop the reaction. In the pretreatment method, samples were centrifuged for 5 min at 12,000 rpm to remove the precipitates, and further filtered through a 0.22 μm pore size membrane. The prepared sample was analyzed by HPLC to quantitatively determine the reaction products. The conversion rates were calculated from the integration of the corresponding HPLC peaks.

Substrate specificity of the *Am*PNP for the phosphorolysis reactions of various purine nucleosides were analyzed and quantified. The phosphorolysis reactions catalyzed by the enzyme toward different purine nucleosides (10 mM) were conducted under 50 °C and pH 8.0 (10 mM Na_2_HPO_4_/KH_2_PO_4_) from 0 h to 48 h. The activity assay was carried out as described in the analysis of enzyme activity, and the highest activity was set to 100%.

### 3.4. Effect of pH on the Activity and Stability of AmPNP

To measure the optimum pH for the phosphorolysis of inosine (10 mM), the activity of the purified *Am*PNP was determined at 50 °C in 10 mM Na_2_HPO_4_/KH_2_PO_4_ buffer with a pH ranging from 5.0 to 8.0. To determine the stability of *Am*PNP under different pH conditions, the purified enzyme was incubated at 50 °C for 2 h. Activity assay was carried out as described in Section 3.3, and the highest activity was set to 100%. Controls without enzymes were performed in parallel.

### 3.5. Effect of Temperature on the Activity and Thermostability of AmPNP

The activity-temperature profile of *Am*PNP was determined from 30 °C to 80 °C (pH 8.0, Na_2_HPO_4_/KH_2_PO_4_) in the presence of the inosine (10 mM) substrate. The thermostability of *Am*PNP was determined by measuring the residual activity at temperatures ranging from 30 °C to 70 °C from 1 to 12 h. The activity assay was carried out as described in Section 3.3, and the highest activity was set to 100%. Controls without enzymes were performed in parallel.

### 3.6. Effect of Temperature and pH on Two-Enzyme Biosynthesis of 2-Amino-6-chloropurine Ribonucleoside *(**2**)*

The reaction mixture (1.0 mL) contained 20 mM uridine and 10 mM 2-amino-6-chloropurine, purified *Bb*PyNP, and purified *Am*PNP. To investigate the effect of temperature on the transglycosylation, the reaction was conducted in a thermostatic oscillator at a pH of 8.0 (20 mM Na_2_HPO_4_/KH_2_PO_4_) with temperatures ranging from 30 °C to 65 °C for 36 h. To determine the optimum pH, the reaction was carried out at a temperature of 55 °C for 36 h in 20 mM Na_2_HPO_4_/KH_2_PO_4_ buffer systems with the pH ranging from 5.0 to 8.0. The products were detected with HPLC in triplicates.

### 3.7. Effect of BbPyNP and AmPNP Ratio on the Biosynthesis of 2-Amino-6-chloropurine Ribonucleoside *(**2**)*

Using 20 mM uridine as the donor, the reaction mixture (1.0 mL) contained purified *Bb*PyNP (85 IU/mg for uridine) and purified *Am*PNP (83 IU/mg) at different ratios (the concentration of *Am*PNP was set at 20 μg/mL, and *Bb*PyNP and *Am*PNP were set with various ratios of 1:1, 0.75: 1, 0.5:1, 0.25: 1, 0.2:1, and 0.1:1, respectively). The reaction was carried out at pH 7.0 and a temperature of 55 °C for 36 h, followed by analysis.

### 3.8. Enzymatic Synthesis of Various Purine Nucleoside Analogs

Using uridine or 2′-deoxyuridine as donor, the one-pot, two-enzyme reaction was carried out with *Bb*PyNP and *Am*PNP (2:20 μg/mL) in 20 mM Na_2_HPO_4_/KH_2_PO_4_ buffer (pH 7.0) and at 55 °C. Different purine bases were used to test the following: 10 mM 2-chloroadenine (2CA), 10 mM 2,6-diaminopurine (DAP), 10 mM 6-thioguanine (2N6SP), 10 mM 6-chloro-2-fluoropurine (6C2FP), 10 mM 2,6-dichloropurine (26DCP), 5 mM 2-fluoroadenine (2FA), 10 mM 2-amino-6-chloropurine (2N6CP), and 5 mM 6-chloropurine (6CP). The reaction mixture was obtained and then analyzed by HPLC to quantitatively determine the reaction products. The pretreatment method was carried out as described in Section 3.3.

### 3.9. HPLC Analysis and Purification and Structural Determination of the Product

The reaction mixtures were analyzed by HPLC coupled with a detector at 254 nm (but 280 nm for nucleosides 7 and 15, and 207 nm for nucleoside 17) and the C18 column (250 × 4.6 mm, 5 μm) at 30 °C. The flow rate was set at 1.0 mL/min, and the injection volume was 5 μL. Gradient elution was done from 0 to 8 min using 95/5 (*v/v*, here and subsequently) water/methanol, from 8 to 9 min with water/methanol changed from 95/5 to 80/20, from 9 to 20 min with a water/methanol ratio of 80/20, from 20 to 22 min with a water/methanol ratio of 80/20, and from 22 to 25 min with a water/methanol ratio of 95/5 to analyze the enzymatic synthesis of purine nucleosides. Elution was performed from 0 to 20 min using only water for the analysis of nucleoside 17. HPLC retention times and HPLC chromatograms of the transglycosylations are given in the Supplementary Materials (Figures S2–S20). The percentage conversion was calculated on the basis of the depletion of the sugar acceptor compound (heterocyclic base) and monitoring the formation of the nucleoside products, with the conversion (%) calculated as [product area/(product area + base area)] × 100%. The product was separated and purified with flash column chromatography using ethyl acetate/methanol (95/5, *v/v*). The structure determination of the products was found by mass spectrometry using a Voyager-DE MALDI-TOFMS apparatus (Applied Biosystems, Franklin Lakes, NJ, USA) and by nuclear magnetic resonance (NMR) using a model (Bruker AV-400 spectrometer (Bruker, Basel, Switzerland).

## 4. Conclusions

In summary, we cloned and characterized a new trimeric PNP from *A. migulanus* AM007, which showed good thermostability and a broad range of pH stability. Then, a one-pot, two-enzyme mode of transglycosylation reactions was successfully constructed by combining *Bb*PyNP and *Am*PNP for the production of purine nucleoside analogs. Mainly, the ratio of *Bb*PyNP and *Am*PNP was optimized in the one-pot, two-enzyme reactions, which greatly reduced the production cost. Highly efficient synthesis of the purine nucleoside analogs was successfully achieved in the one-pot, two-enzyme transglycosylation reactions catalyzed by *Am*PNP and *Bb*PyNP for the higher concentrations of substrates (pentofuranosyl donor: purine base; 20:10 mM) with a low enzyme ratio of *Bb*PyNP: *Am*PNP (2:20 μg/mL). Therefore, the newly trimeric *Am*PNP is a promising biocatalyst for the industrial production of purine nucleoside analogs.

## Figures and Tables

**Table 1:**
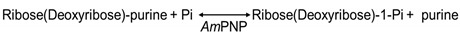
Substrate specificity of *Am*PNP.

Substrate	Phosphorolysis (%)	Relative Activity (%)
Inosine	32.7 ± 3.9	100 ± 1.5
Guanosine	21.9 ± 4.9	69.7 ± 4.8
Adenosine	9.5 ± 2.1	3.1 ± 1.7
2′-Deoxyadenosine	0	0
2′-Deoxyguanosine	16.2 ± 1.3	17.2 ± 4.2

The phosphorolysis reactions catalyzed by the enzyme toward different purine nucleosides (10 mM) were conducted under 50 °C and pH 8.0 (10 mM Na_2_HPO_4_/KH_2_PO_4_) from 0 h to 48 h. Samples were taken every 5 min, and 50 μL of the mixture was added to 950 μL methanol for reaction termination. Data are represented as mean ± SD (n = 3). Equilibrium was reached in the applied reactions and phosphorolysis (%) was absolute conversion (%). Maximum enzyme activity of *Am*PNP was 83 IU/mg for inosine and relative activity was set 100%.

**Table 2:** Kinetic parameters for *Am*PNP with various purine nucleosides.

Substrate	K_m_ (mol/L)	K_cat_ (s^−1^)	K_cat_/K_m_ (s^−1^ M^−1^)
Inosine	5.30 × 10^−3^	6.38 × 10^4^	1.21 × 10^7^
Guanosine	1.16 × 10^−3^	1.84 × 10^4^	1.58 × 10^7^
Adenosine	1.45 × 10^−3^	1.43 × 10^1^	9.92 × 10^3^

In total, 1 mL of reaction mixture was composed of 10 mM sodium phosphate buffer (pH 8.0), an appropriate amount of *Am*PNP, and 0.5–5 mM substrates. The reaction mixture was incubated at 50 °C. Samples were taken every 5 min, and 50 μL of the mixture was added to 950 μL methanol for reaction termination.

**Table 3:**
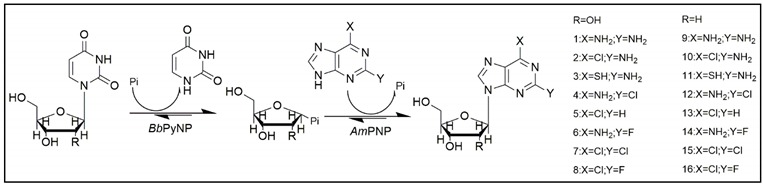
Biosynthesis of purine nucleoside analogs.

Entry	Pentofuranosyl Donor	Purine Base		Products	Ribosylation	Time
	R	X	Y	Nucleoside	(%)	(h)
1	Uridine OH	NH_2_	NH_2_	DAP-R (**1**)	98.3	24
2		Cl	NH_2_	2N6CP-R (**2**)	95.1	36
3		SH	NH_2_	2N6SP-R (**3**)	92.3	4
4		NH_2_	Cl	2C-Ado (**4**)	81.6	48
5		Cl	H	6CP-R (**5**)	47.7	36
6		NH_2_	F	2F-Adeo (**6**)	43.9	48
7		Cl	Cl	26DCP-R (**7**)	6.6	48
8		Cl	F	2F6CP-R (**8**)	3.4	48
9	2′-Deoxyuridine H	NH_2_	NH_2_	DAP-dR (**9**)	96.5	24
10		Cl	NH_2_	2N6CP-dR (**10**)	74.1	36
11		SH	NH_2_	2N6SP-dR (**11**)	89.3	4
12		NH_2_	Cl	Cladribine (**12**)	60.4	48
13		Cl	H	6CP-dR (**13**)	65.8	36
14		NH_2_	F	2F-Adeo (**14**)	28.3	48
15		Cl	Cl	26DCP-dR (**15**)	7.3	48
16		Cl	F	2F6CP-dR (**16**)	8.2	48
17	Uridine	1,2,4-Triazole-3-carboxylicacid		Ribavirin (**17**)	73.7	12

Temperature: 55 °C. Enzymes: *Bb*PyNP: *Am*PNP. 2:20 μg/mL; Medium: 20 mM Na_2_HPO_4_/KH_2_PO_4_ buffer (pH 7.0); Substrates: pentofuranosyl donor: Purine base. 20:10 mM (entries 1–3, 5, 7–11, 13, 15–17); 10: 5 mM (entries 4, 6, 12, 14).

## Supplementary Materials

The following are available online, Figure S1: Effect of proportion of glycosyl donor and purine base on the biosynthesis of 2-amino-6-chloropurine ribonucleoside, Figures S2–S20: HPLC dates of synthesis of various purine nucleoside analogs, Figure S21: 1H-NMR spectrum of 2-amino-6-chloropurine nucleoside, Figure S22: 13C-NMR spectrum of 2-amino-6-chloropurine nucleoside, Table S1: Identities of the reported PNPs, Table S2: Half-life of *Am*PNP at different temperature.
