# Supplementary material for: Efficient Synthesis of Purine Nucleoside Analogs by a New Trimeric Purine Nucleoside Phosphorylase from Aneurinibacillus migulanus AM007
Source: Molecules. 2019 Dec 26;25(1):100. doi: 10.3390/molecules25010100 (PMC6983109; doi:10.3390/molecules25010100)
Supplement: Supplementary file 1 [file molecules-25-00100-s001.pdf]

Supplementary Materials

Efficient synthesis of nucleoside analogues by a new thermostable purine nucleoside phosphorylase from *Aneurinibacillus migulanus* AM007

Gaofei Liu[a], Tiantong Cheng[a], Jianlin Chu[b, *], Sui Li[b], Bingfang He[b, *]

[a] College of Biotechnology and Pharmaceutical Engineering, Nanjing Tech University, Nanjing 211800, China

[b] School of Pharmaceutical Sciences, Nanjing Tech University, Nanjing 211800, China

*Corresponding authors: E-mail: bingfanghe@njtech.edu.cn; cjl2fl@126.com

Table.S1 Identities of the reported PNPs

Trimer	Source	Identity	Hexamer	Source	Identity（%）
HsPNP	Homo sapiens	46.4	*Ec*PNP	*Escherichia coli*	14.7
MmPNP	Mus musculus	45.3	*Ah*PNP	*Aeromonas hydrophila*	13.1
BtPNP	Bos taurus	45.3	*Kp*PNP	*Klebsiella sp*	13.7
GivPNP	*Grouper iridovirus*	45.3	*Tt*6PNP	*Thermus thermophilus*	16.7
*Tt*3PNP	*Thermus thermophilus*	49.8	*St*PNP	*Streptococcus thermophilus*	17.9
*Bs*PNP	*Bacillus subtilis*	66.1	*Ss*PNP	*Sulfolobus solfataricus*	13.2

HsPNP (GenBank: NP_000261); MmPNP (GenBank: NP_038660); BtPNP (GenBank: AAX46392); GivPNP (GenBank: AY598033); *Tt*3PNP (GenBank: BAD70385); *Bs*PNP (GenBank: WP_015714207); *Ec*PNP (GenBank: AAN83888); *Ah*PNP (GenBank: WP_049045821); *Kp*PNP (GenBank: CAA61136); *Tt*6PNP (GenBank: WP_096412123); *St*PNP (GenBank: WP_011681178); *Ss*PNP (GenBank: WP_009988635). The identities were calculated by the amino acid sequences of PNPs.

Table. S2 Half-life of *Am*PNP at different temperature

Temperature (°C)	Half-life (h)
50	162.2 ± 3.8
55	78.6 ± 4.3
60	37.3 ± 2.8
65	6.9 ± 0.3
70	0.4 ± 0.1

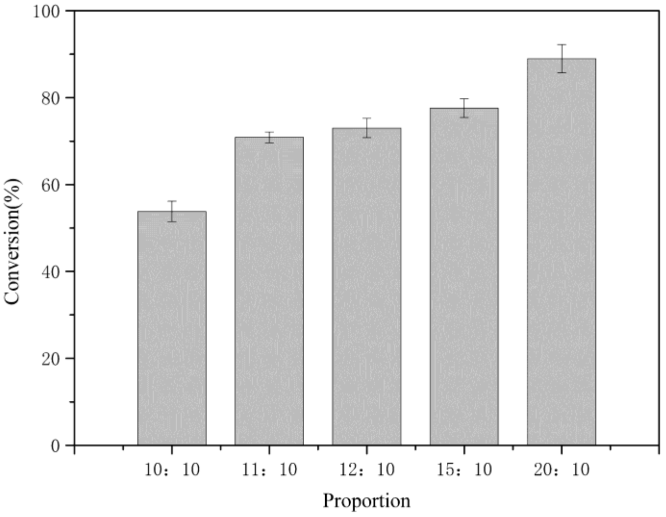

Fig. S1 Effect of proportion of glycosyl donor and purine base on the biosynthesis of 2-amino-6-chloropurine ribonucleoside

The proportions of uridine donor and 2-amino-6-chloropurine base were set at 10 mM:10 mM, 11 mM:10 mM, 12 mM:10 mM, 15 mM:10 mM and 20 mM:10 mM, respectively.

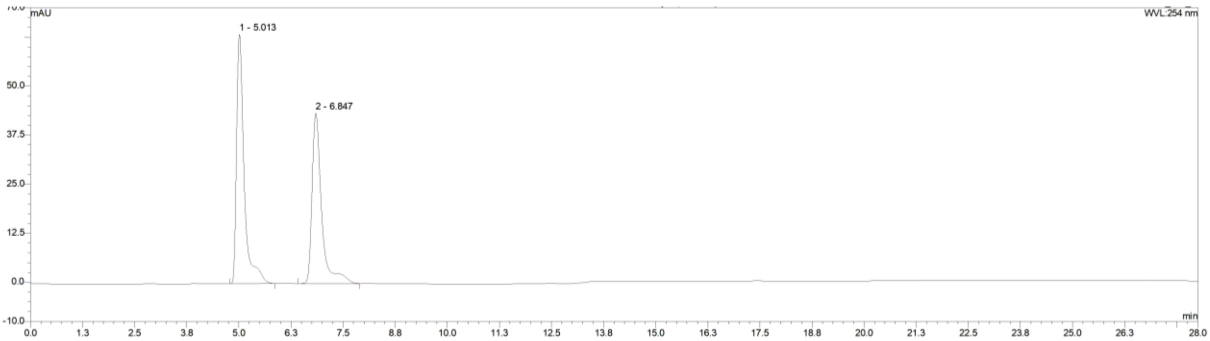

Fig. S2. HPLC analysis of the phosphorolysis activity of *Am*PNP toward uridine.

Ura (Rt=5.013 min), Urd (Rt=6.847 min)

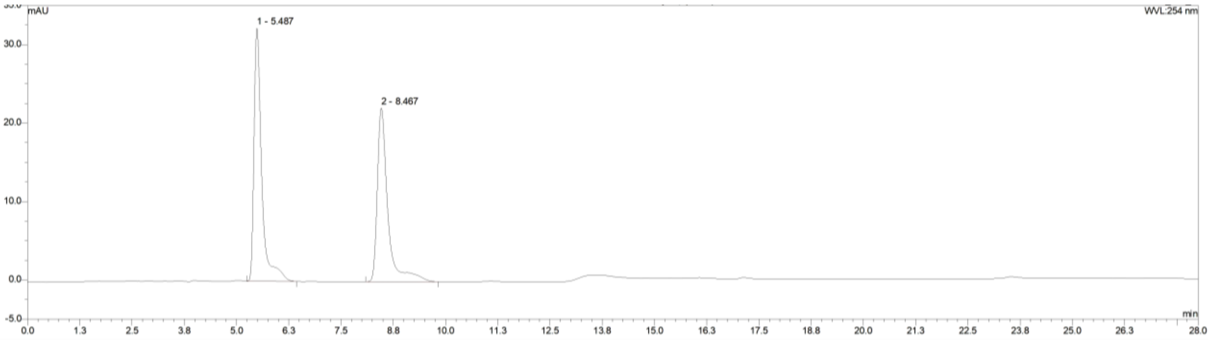

Fig. S3. HPLC analysis of the phosphorolysis activity of *Am*PNP toward 2'-Deoxyuridine.

Ura (Rt=5.487 min), 2'-dU (Rt=8.467 min)

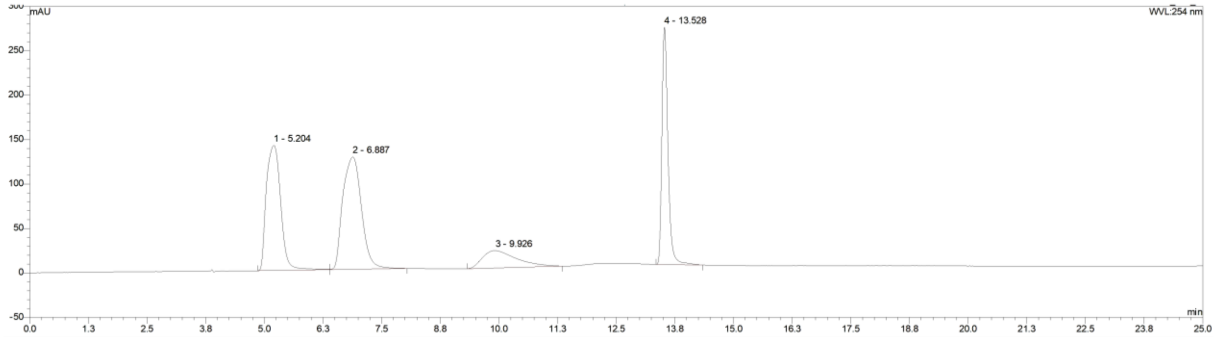

Fig. S4. Enzymatic synthesis of DAP-R (1) by HPLC analysis.

Ura (Rt=5.204 min), Urd (Rt=6.887 min), DAP (Rt=9.926 min), DAP-R (Rt=13.528 min)

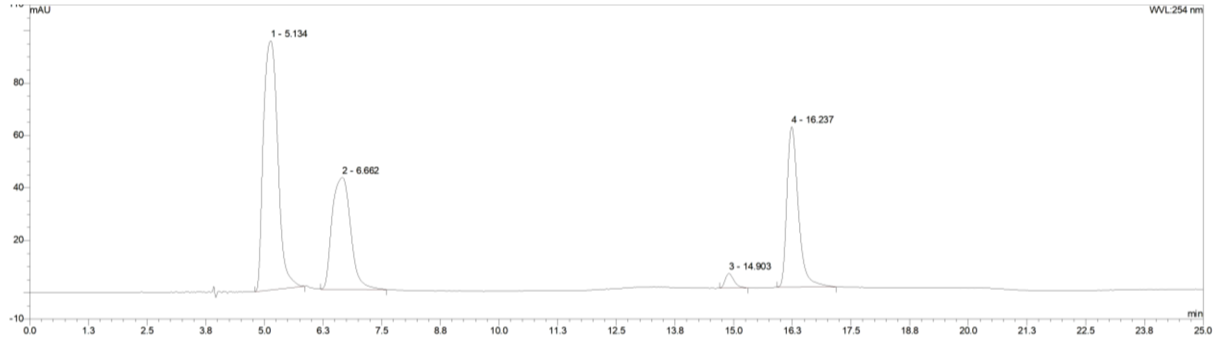

Fig. S5. Enzymatic synthesis of 2N6CP-R (2) by HPLC analysis.

Ura (Rt=5.134 min), Urd (Rt=6.662 min), 2N6CP (Rt=14.903 min), 2N6CP-R(Rt=16.237 min)

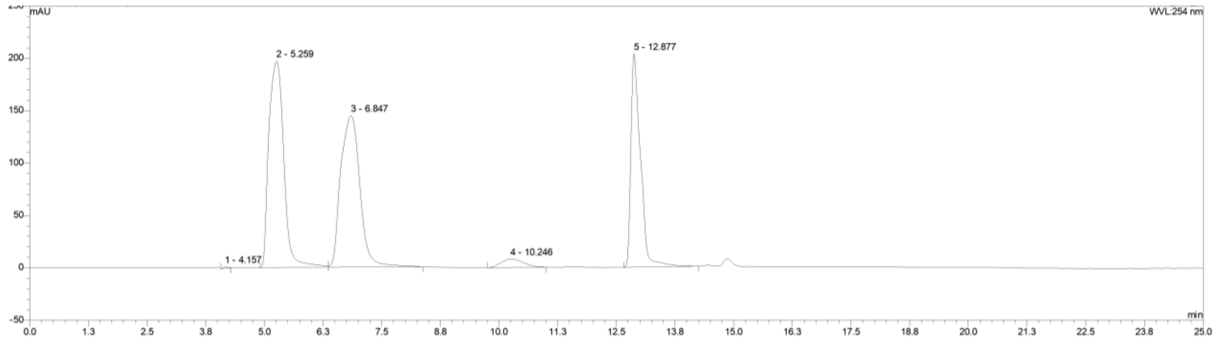

Fig. S6. Enzymatic synthesis of 2N6SP-R (3) by HPLC analysis.

Ura (Rt=5.259 min), Urd (Rt=6.847 min), 2N6SP (Rt=10.246 min), 2N6SP-R (Rt=12.877 min)

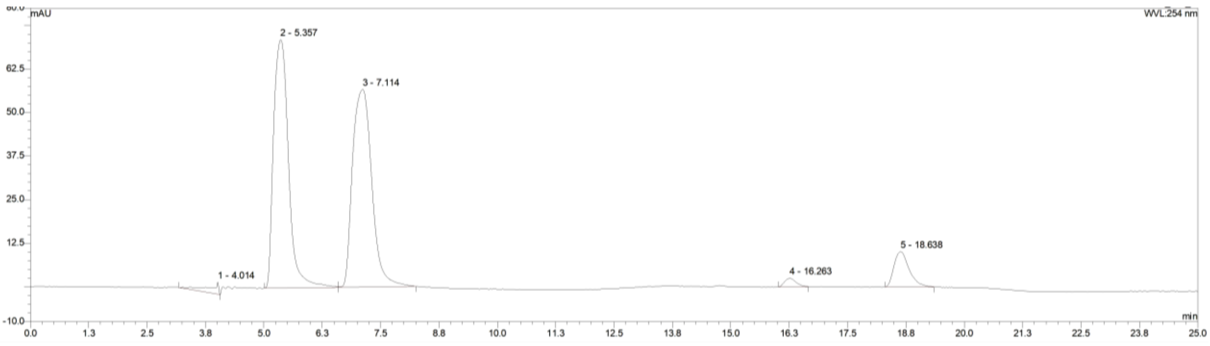

Fig. S7. Enzymatic synthesis of 2C-Ado (4) by HPLC analysis.

Ura (Rt=5.357 min), Urd (Rt=7.114 min), 2CA (Rt=16.263 min), 2C-Ado (Rt=18.638 min)

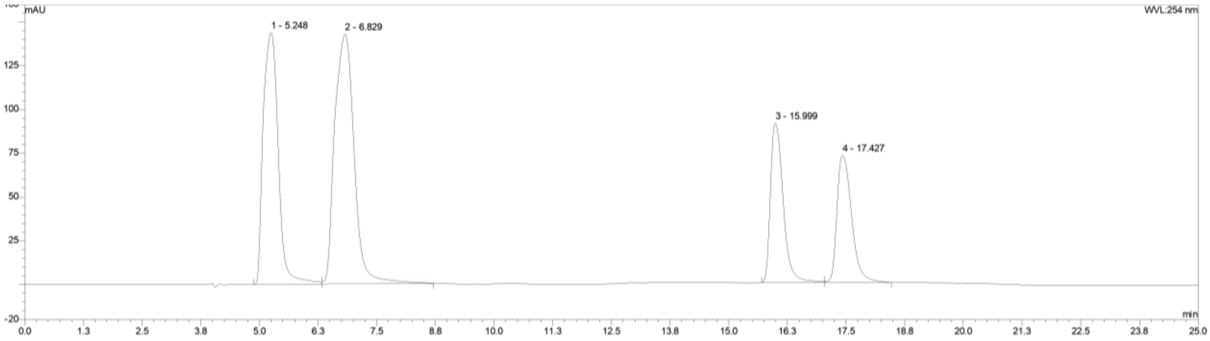

Fig. S8. Enzymatic synthesis of 6CP-R (5) by HPLC analysis.

Ura (Rt=5.248 min), Urd (Rt=6.829 min), 6CP (Rt=15.999 min), 6CP-R (Rt=17.427 min)

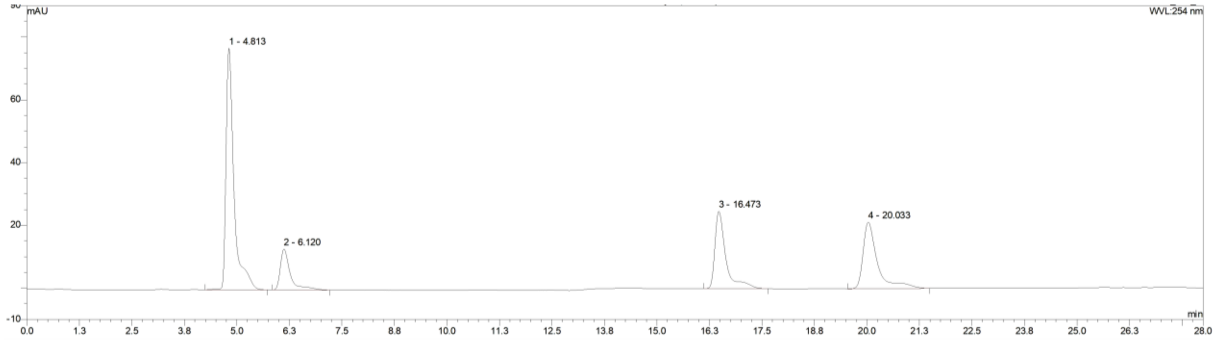

Fig. S9. Enzymatic synthesis of 2F-Ado (6) by HPLC analysis.

Ura (Rt=4.733 min), Urd (Rt=5.773 min), 2FA (Rt=15.713 min), 2F-Ado (Rt=18.993 min)

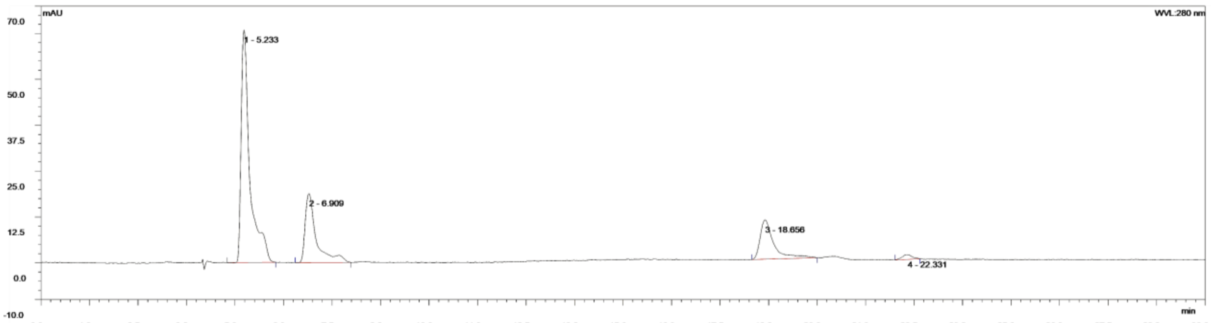

Fig. S10. Enzymatic synthesis of 26DCP-R (7) by HPLC analysis.

Ura (Rt=5.233 min), Urd (Rt=6.909 min), 26DCP (Rt=18.656 min), 26DCP-R (Rt=22.334 min)

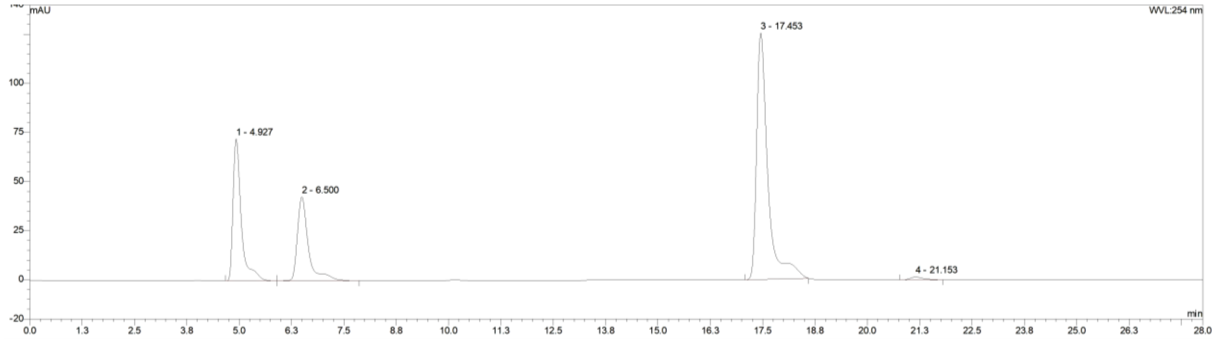

Fig. S11. Enzymatic synthesis of 2F6CP-R (8) by HPLC analysis.

Ura (Rt=4.927 min), Urd (Rt=6.500 min), 2F6CP (Rt=17.453 min), 2F6CP-R（Rt=21.153 min）

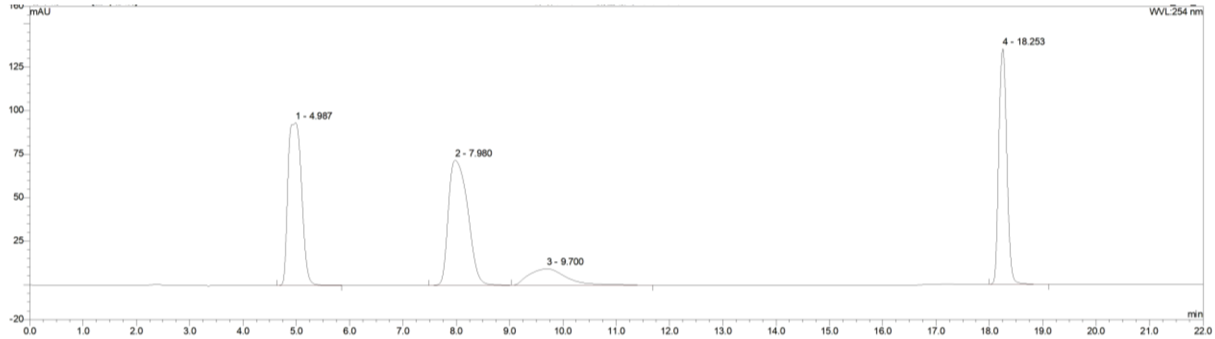

Fig. S12. Enzymatic synthesis of DAP-dR (9) by HPLC analysis.

Ura (Rt=4.987 min), 2'-dU (Rt=7.980 min), DAP (Rt=9.700 min), DAP-dR (Rt=18.253 min)

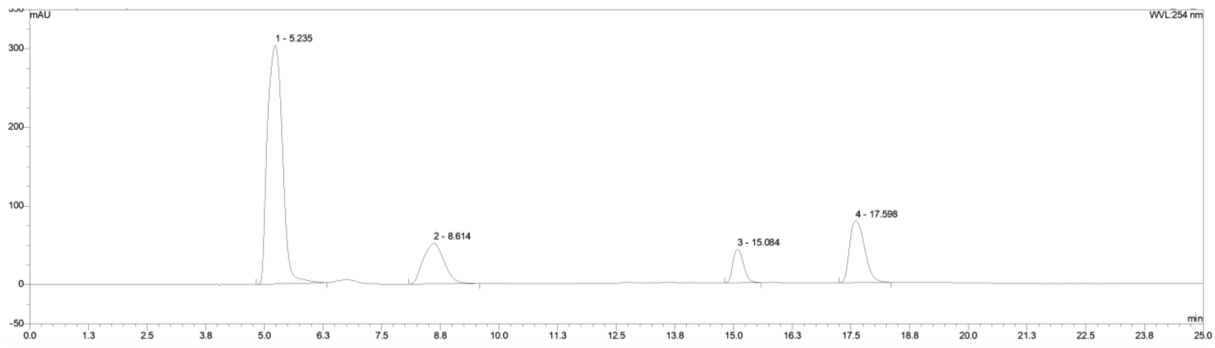

Fig. S13. Enzymatic synthesis of 2N6CP-dR (10) by HPLC analysis.

Ura (Rt=5.235 min), 2'-dU (Rt=8.614 min), 2N6CP (Rt=15.084 min), 2N6CP-dR (Rt=17.598 min)

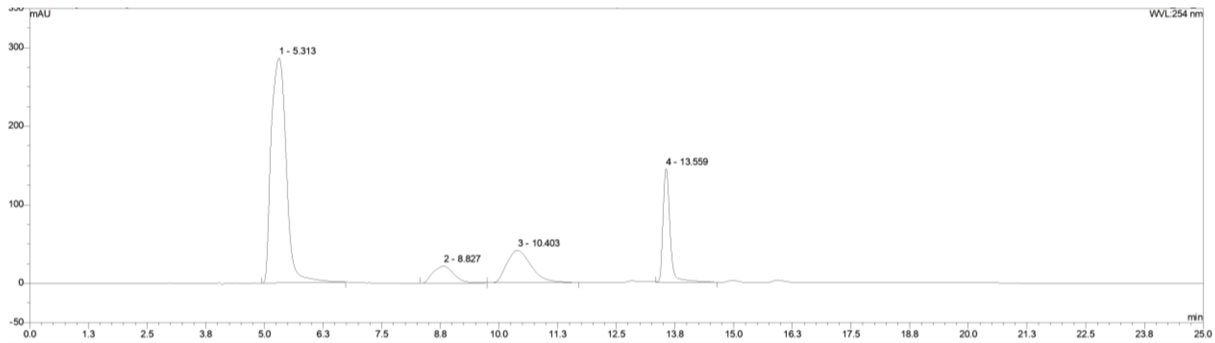

Fig. S14. Enzymatic synthesis of 2N6SP-dR (11) by HPLC analysis.

Ura (Rt=5.313 min), 2'-dU (Rt=8.827 min), 2N6SP (Rt=10.403 min), 2N6SP-dR (Rt=13.559 min)

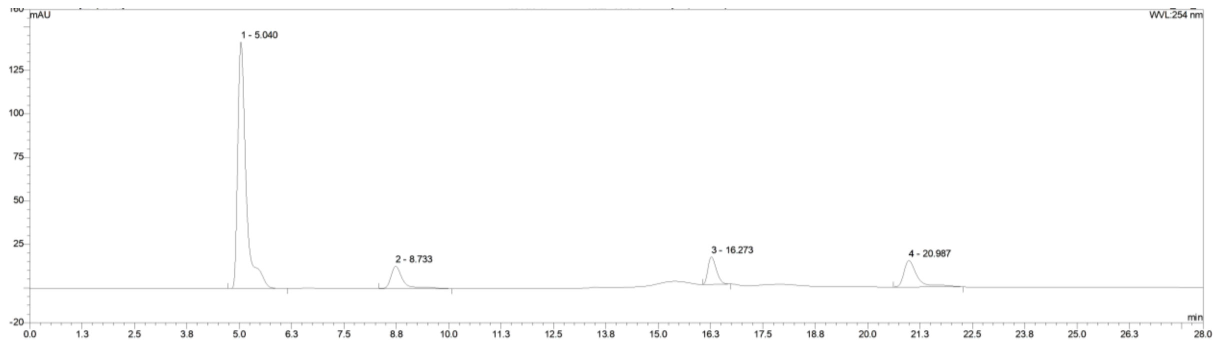

Fig. S15. Enzymatic synthesis of Cladribine (12) by HPLC analysis.

Ura (Rt=5.040 min), 2'-dU (Rt=8.733 min), 2CA (Rt=16.273 min), Cladribine (Rt=20.987 min)

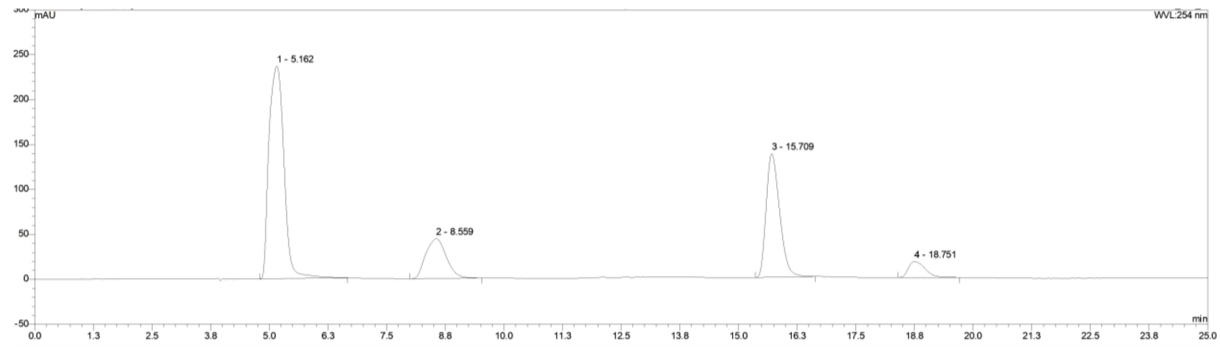

Fig. S16. Enzymatic synthesis of 6CP-dR (13) by HPLC analysis.

Ura (Rt=5.162 min), 2'-dU (Rt=8.559 min), 6CP (Rt=15.709 min), 6CP-dR (Rt=18.751 min)

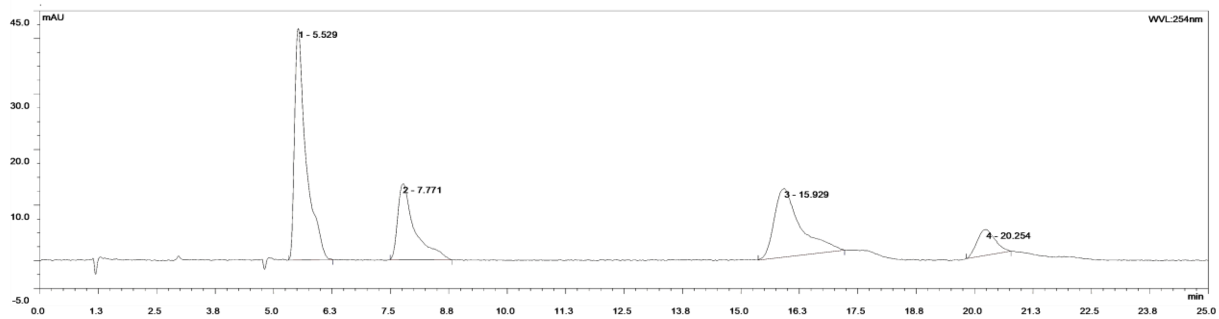

Fig. S17. Enzymatic synthesis of 2F-dAdo (14) by HPLC analysis.

Ura (Rt=5.529 min), 2'-dU (Rt=7.771 min), 2FA (Rt=15.929 min), 2F-dAdo (Rt=20.254 min)

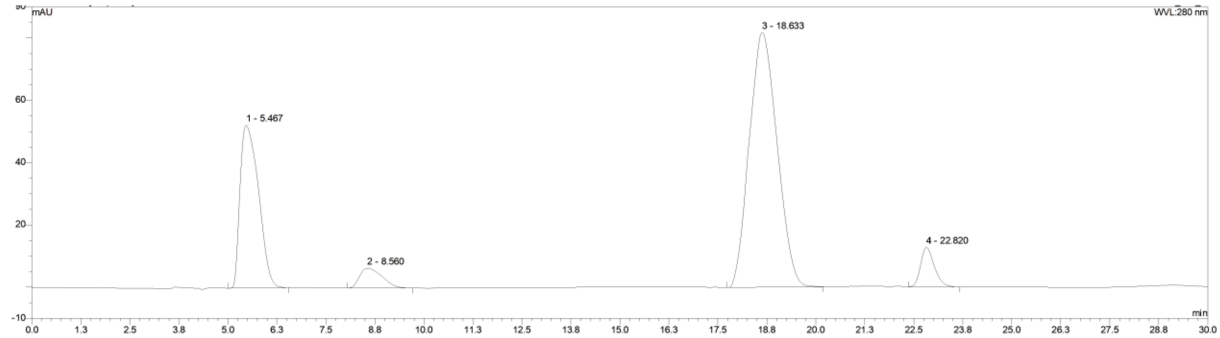

Fig. S18. Enzymatic synthesis of 26DCP-dR (15) by HPLC analysis.

Ura (Rt=5.467 min), 2'-dU (Rt=8.560 min), 26DCP (Rt=18.633 min), 26DCP-dR (Rt=22.820 min)

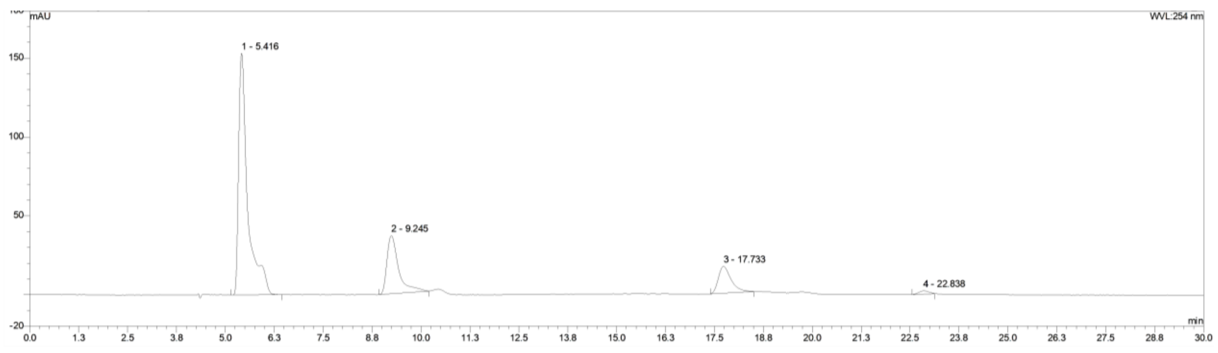

Fig. S19. Enzymatic synthesis of 2F6CP-dR (16) by HPLC analysis.

Ura (Rt=5.416 min), 2'-dU (Rt=9.245 min), 2F6CP (Rt=17.733 min), 2F6CP-dR (Rt=22.838 min)

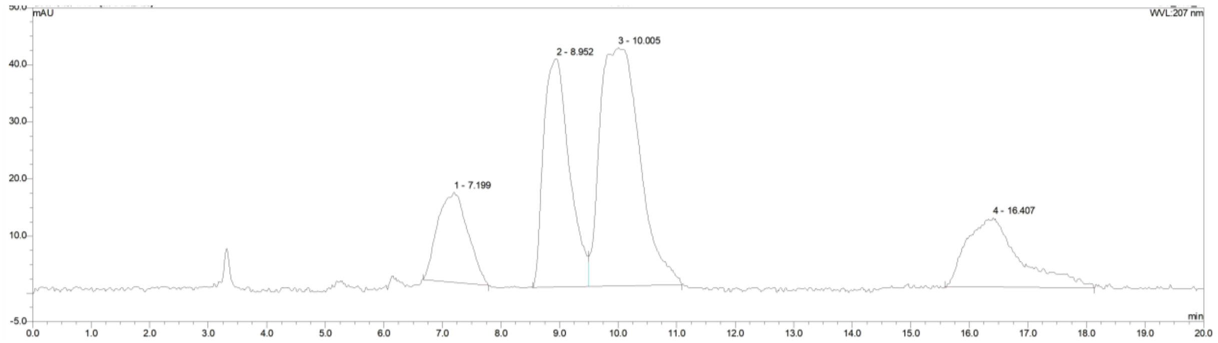

Fig. S20. Enzymatic synthesis of Ribavirin (17) by HPLC analysis.

Ura(Rt=8.952 min), Urd(Rt=16.407 min), 1,2,4-Triazole-3-carboxylicacid (Rt=7.199 min), Ribavirin (Rt=10.005 min)

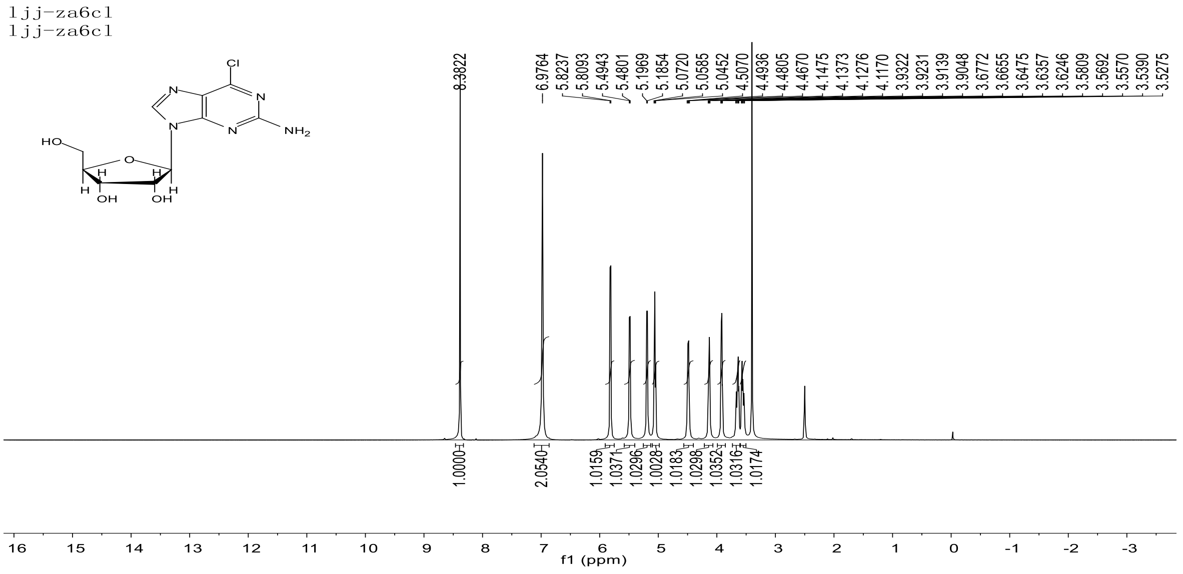

Fig. S21. ^{1}H-NMR spectrum of 2-amino-6-chloropurine nucleoside (2)

^{1}H-NMR (400 MHz, DMSO-*d6*) *δ*/ppm 8.38 (s, 1H), 6.98 (s, 2H), 5.82 (d, *J* = 5.7 Hz, 1H), 5.49 (d, *J* = 5.7 Hz, 1H), 5.19 (d, *J* = 4.6 Hz, 1H), 5.06 (t, *J* = 5.4 Hz, 1H), 4.49 (dd, *J* = 10.6, 5.4 Hz, 1H), 4.13 (dd, *J* = 8.1,

4.2 Hz, 1H), 3.92 (dd, J = 7.3, 3.6 Hz, 1H), 3.65 (dt, J = 9.2, 4.6 Hz, 1H), 3.50 -3.60 (m, 1H).

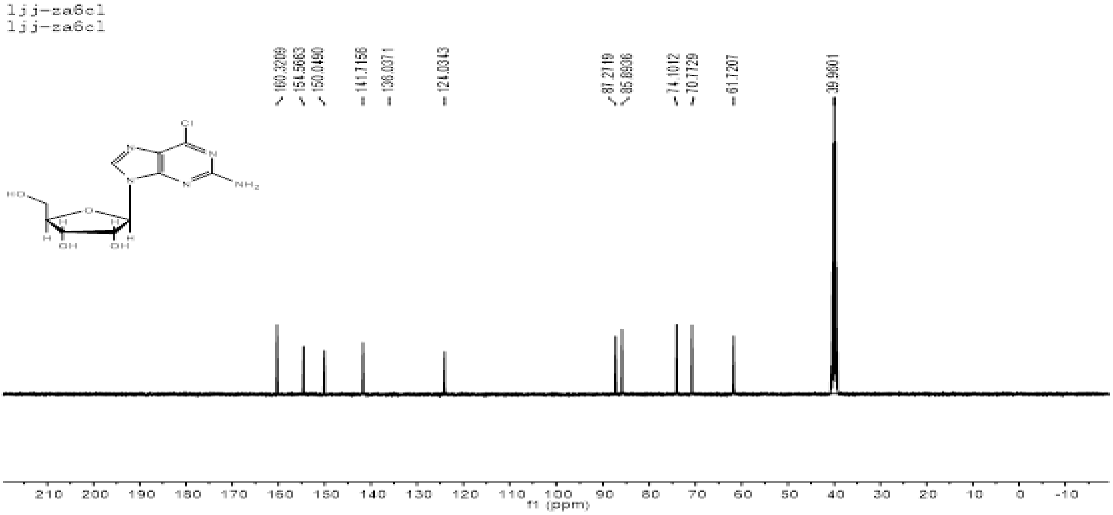

Fig. S22. ^{13}C-NMR spectrum of 2-amino-6-chloropurine nucleoside (2)

^{13}C-NMR (100 MHz, DMSO-*d6*) δ/ppm 160.32, 154.57, 150.05, 141.72, 124.03, 87.27, 85.89, 74.10, 70.77, 61.72.
